# The effect of regional transmural agreements on the information transfer of frail older patients

**DOI:** 10.1186/s12877-023-04519-4

**Published:** 2023-11-29

**Authors:** G. Fritsche, N. Schoonenboom, H. Van der Kroon, CE. Douma, J. Van der Dussen, MNE. Verlaan, B. Cloosterman, M. Heems, A. Nepal, E. J. Toor, A. de Rooij, KJ. van Stralen, JA. Lucke

**Affiliations:** 1 Department of Emergency Medicine, Spaarne Gasthuis, Boerhaavelaan 22, Haarlem, 2035 RC The Netherlands; 2 Department of Neurology, Spaarne Gasthuis, Haarlem, The Netherlands; 3 Department of Geriatrics, Spaarne Gasthuis, Haarlem, The Netherlands; 4 Department of Internal Medicine, Spaarne Gasthuis, Haarlem, The Netherlands; 5 General Practitioner, Rijsenhout, The Netherlands; 6 General Practitioner, Haarlem, The Netherlands; 7 General Practitioner, Badhoevedorp, the Netherlands; 8 Elderly Care Physician, Amstelring, Hoofddorp, The Netherlands; 9 Elderly Care Physician, Zorgbalans, Haarlem, The Netherlands; 10 Transmural Coordinating Centre, Spaarne Gasthuis, Haarlem, The Netherlands; 11 Spaarne Gasthuis Academy, Spaarne Gasthuis, Hoofddorp, The Netherlands

**Keywords:** Emergency Department, General Practitioner, ECP nursing home, Information transfer, Frail older patient

## Abstract

**Introduction:**

Frail older patients are at risk for many complications when admitted to the hospital. Multidisciplinary regional transmural agreements (RTA) in which guidelines were set concerning the information transfer of frail older patients might improve outcomes. We aim to investigate the effect of implementation of the RTA on the completeness of the information transfer of frail older patients when admitted to and discharged from the hospital.

**Methods:**

This is a retrospective cohort study in which we collected data from 400 randomly selected hospitalized frail older patients (70+) before the implementation of the RTA, January through March 2021, and after, October through December 2021. The cohort was split up into four groups, which determined what correspondence would be checked (referral letter by General Practitioner (GP) and three groups of ‘hospital letters’: ED letter upon admittance, clinical discharge letter to the elderly care physician and clinical discharge letter to the GP. We assessed for mention of frailty, a medication list and mention of resuscitation orders.

**Results:**

In the period before implementation the mean age of patients was 82.6 years (SD 7.4) and 101 were female (50.5%), after implementation mean age was 82.3 (SD 6.9) and 112 were female (56.0%). Frailty was mentioned in hospital letters in 12.7% before and 15.3% after implementation (*p* = 0.09). More GP referral letters were present after implementation (32.0% vs. 54.0%, *p* = 0.03), however frailty was mentioned only in 12.5% before and 7.4% after (*p* = 0.58). There was a good handover of medication lists from the hospital (89.3% before, 94% after, *p* = 0.20) and even better from the GP (93.8% before, 100% after, *p* = 0.19). Resuscitation orders were mentioned in 59.3% of letters from the hospital before implementation and 57.3% after (*p* = 0.77), which is higher than in the referral letters (18.8% before and 22.2% after (*p* = 0.91).

**Discussion:**

The implementation of RTA improved the number of GP referral letters present; however, it did not lead to other significant improvements in communication between the hospital and the GP’s. Frailty and resuscitation orders are still frequently not mentioned in the reports. After a successful reimplementation, the improvements of outcomes could be investigated.

**Supplementary Information:**

The online version contains supplementary material available at 10.1186/s12877-023-04519-4.

## Background

Older patients who are hospitalized are at risk for complications, such as functional decline and decreased quality of life [1–4]. Frailty impacts the risk of hospitalization, falls and mortality [5, 6]. Older patients can be hospitalized because of a geriatric syndrome or develop a geriatric syndrome during or shortly after their hospital stay [7] and these increase morbidity and mortality risks. If patients are discharged, information about frailty, advanced care directives and medication use is very important to their General Practitioner (GP) or elderly care physician (ECP). Although the transfer of medical information of patients throughout the health care system is very important, it is not always complete or accurate [8, 9]. This is an added risk for frail older patients. Therefore, protocols to improve information transfer, and thus reduce risks associated with information loss, are important [10, 11].

To improve care for older patients a multidisciplinary transmural project was started to make guidelines with regards to the information transfer of frail older patients before admittance and after discharge from the hospital. Regional transmural agreements (RTA) [12] can be a way to formalize agreements among different healthcare providers to provide a continuum of care [13]. RTA’s are a model that is increasingly used in the Netherlands, in many different fields of medicine such as chronic heart failure, wound care and palliative care, and has been evaluated before. However the collaboration between primary and secondary care in these projects is not always successful [14]. Adherence to these agreements might depend on numerous factors such as adherence by the staff, attitude towards change, underlying knowledge and ease of use [15, 16]. Therefore, it is important to evaluate whether implementing the RTA has been successful or further steps on implementation and collaboration should be made.

This study aims to see whether the RTA that was implemented influenced the medical information transfer concerning frail older people, between care professionals. This is important, because it will show the success of implementing RTA’s with the chosen strategy, as a first step towards improving patient care.

## Methods

### Study design and setting

This is a retrospective cohort study, with patients included before and after implementation of the RTA on May 3, 2021. The data for this study was collected between January through March 2021, before the implementation, and between October through December 2021, after the implementation. The participants are patients of the Spaarne Gasthuis hospital, a large 200 bed teaching hospital in the Netherlands.

The RTA was an initiative of a transmural collaboration bureau called ‘Medical Coordinating Centre ‘Haarlem en Meer’’ which mission it is to improve the collaboration between GP’s, hospital and ECP’s. The mobile phone app called ‘NHZ-connected’ was created to improve transmural communications and make it simpler to find the RTA’s on several topics and contains contact information of all the GP’s, ECP’s and medical specialists in the region in order to improve communication [12]. This RTA specifically focused on frail older patients and was developed by a working group consisting of GP’s, ECPs, and medical specialists. The agreements included requirements for correspondence, such as including information on frailty, medical history, medication lists, and resuscitation orders. Additionally, GP’s were expected to identify and record frailty in the medical records of older patients, while emergency physicians were instructed to consider the information provided by GP’s.

The goal of the RTA was to ensure seamless continuity of care, reduce unplanned readmissions, and improve the satisfaction of care professionals regarding information transfer. To facilitate successful implementation, posters, a video, and an instruction handbook for new doctors were utilized. Furthermore, guidelines were presented during visits to each department. GP’s were informed through newsletters, an animation video, and mouse pads, and a presentation was delivered at a regional meeting for GP’s.

### Study participants – inclusion and exclusion criteria

We included frail patients aged 70 years or older who were admitted to our hospital. This age cut-off was used as this is similar to the safety management system ‘frail older patients’, which is mandatory by the Dutch Health and Youth Care Inspectorate (VMS (veiligheidsmanagement systeem) programma ‘Kwetsbare oudere’ [17]) to define a ‘older person’. At time of inclusion, the patients needed to meet the criteria of frailty defined as the occurrence of one of the items of the VMS [17] being positive or the Clinical Frailty Scale (CFS) [18] being 5 or higher (CFS only applies to ‘after implementation’ group). Patients from all departments could be included. Patients who deceased in-hospital were excluded from this study. Patients with missing correspondence (either referral letter or discharge letters) were not excluded to reduce risk of selection bias. Between January and March 1358 frail older patients were hospitalized and between October and December 1417 patients. From both periods we included a computerized random sample of 200 patients, leading to a sample of approximately 14% of eligible patients.

### Data collection and measurements

The data collection took place retrospectively in the above-mentioned periods. The data was collected from the patient files by a trained medical student (GF) and checked for validity by their supervisor (JAL).

The patients were divided into 4 groups, and in each group a different type of letter was assessed: referral letter from the GP and three groups of ‘hospital letters’; emergency department letter upon admittance, clinical discharge letter to the ECP when admitted to a nursing home and clinical discharge letter to the GP. Of all selected patients the letters were checked for the mention of frailty, the medication list and resuscitation orders.

In total the medical student checked 400 patients’ records, 50 in each category before and after implementation. Additionally, it was checked whether the 200 clinical discharge letters (to the GP and ECP) were sent timely, which is within 24 h, and whether the contained the automated medication prescription list.

#### Frailty scales

Together with the implementation of the RTA, screening for frailty by use of the Clinical Frailty Scale (CFS) in older ED patients was implemented. Before implementation frailty was assessed by measuring the VMS scale [17] in hospitalized patients only, as is mandatory by the Dutch Health and Youth Care inspectorate. The VMS scale is based on delirium, falling, malnutrition and loss of function. If one category is positive, it gives a point with a range of 0–4 points, with one point the patient is considered frail. The delirium screening within VMS contains of three questions: do you have memory problems, did you require help with self-care in the past 24 h, did you ever have delirium before. If one question is answered with yes, it is a positive screening and adds one point to the VMS score. The risk of falling is assessed with the question: did the patient fall one or more times in the past six months, if this is answered with yes, it is a positive screening and adds one point to the VMS score. The risk of malnutrition is assessed with three questions (SNAQ score [19]): did you unintentionally lose weight, did you have a decreased appetite in the last month, did you use medical nutrition or tube feeding in the last month. If one question is answered with yes, it is a positive screening and adds one point to the VMS score. The loss of function is assessed using the Katz-ADL 6 [20] and contains six questions: do you need help with bathing or showering, do you need help dressing, do you need help going to the restroom, do you use incontinence materials, do you need help to transfer from bed to chair, do you need help walking. If one question is answered with yes, it is a positive screening and adds one point to the VMS score.

After the implementation, the CFS was used in ED patients, as well as the VMS for hospitalized patients. The CFS scales measure from a score of 1, very fit, to a score of 9, terminally ill [21]. A patient is considered frail if the score is 5 or higher. The CFS was therefore only available in patients included after implementation.

#### Primary outcome

The letters before and after the implementation of the RTA were assessed for differences in the mentioning of frailty, a medication list and resuscitation orders.

Mention of frailty was scored as complete when either the CFS was mentioned or all the four VMS items, it was scored as incomplete when less than four VMS items were mentioned, for example only risk of delirium.

The medication list was registered as incomplete when for example dosages or frequency of intake were not mentioned.

Resuscitation orders mean mentioning these orders in the correspondence, for example: patient wants to be fully resuscitated, does not want to be resuscitated, does not want to be admitted to the ICU, only wants non-invasive ventilation etc. It was scored as incomplete when patients had a non-ICU wish, but no specific comments were made about non-invasive ventilation.

#### Secondary outcome

The first secondary outcome was whether the discharge letters were sent within the required 24 h of discharge. This was determined by comparing the time of discharge with the time the letter was sent, allowing for an evaluation of the timeliness of the letters. The second secondary outcome was whether the medication list was filled out using the automated prescribing system of our hospital, which has an automatic link to the patient’s own pharmacy and therefore lowering the chance of prescription errors.

### Statistical analysis

SPSS (IBM SPSS statistics version 27) was used to analyze the data. Data is presented as mean with standard deviation when normally distributed for continuous variables, or as median with standard deviation when non-normally distributed and as number with percentages for categorical variables. The mention of frailty, a medication list and resuscitation orders all had polytomous (absent = 0, complete = 1, incomplete = 2) outcomes and were scored using a predefined system. The discharge letters being sent within 24 h and presence of automated medication list had a dichotomous (present = 1, absent = 0) outcome. The Pearson’s Chi-square test was performed for all categorical variables. In a sensitivity analysis, the relationship between level of frailty and handover of frailty in the letters was assessed using a Pearson’s Chi-square test. The number of missing values is showed in the tables, data was not imputed. *P*-values below 0.05 were considered significant.

### Ethics approval and consent to participate

All methods were carried out in accordance with relevant regulations and guidelines. This study was approved by the local institution review board (study number 2022.0007) of the Spaarne Gasthuis. According to Dutch Law (General Data Protection Regulation, grounds for exception - ‘AVG uitzonderingsgronden’) informed consent from the patient was not required as data was collected as part of evaluation of quality of care and all data was handled anonymously. This research does not fall under the ‘Medical research with human subjects law’. Patients are informed on the hospital website that retrospective data can be anonymously used for care evaluation and an opt-out option. This study complied with the declaration of Helsinki.

## Results

### Baseline characteristics

We included 400 patients, of whom 200 patients before and 200 after the implementation (Table 1). Before implementation mean age was 82.6 years (SD 7.4) and 101 patients were female (50.5%), after implementation mean age was 82.3 years (SD 6.9). Most patients were admitted to the departments of geriatrics (n = 57, 28.5% before, n = 50, 25% after), internal medicine (n = 49, 24.5% before, n = 47, 23.5% after) or surgery (n = 30, 15% before, n = 17, 8.5% after). Patients had a mean VMS score of 2.2 (SD 1.0) before and 2.1 (SD 1.0) after the implementation. The CFS was only available after the implementation and the mean score was 5.1 (SD 1.7). Based on the VMS score 86% of patients had increased risk of developing delirium, 46% had risk of falling and 28% had risk of malnutrition in the before group, the after-implementation group had similar risks.

**Table 1:** Baseline characteristics of included patients

	Before implementation n = 200	After implementation n = 200
• Female (n,%)	101 (50.5)	112 (56.0)
• Age (mean, SD)	82.6 (7.4)	82.3 (6.9)
Type of letter reviewed (n,%)		
• Referral*	50 (25.0)	50 (25.0)
• ED**	50 (25.0)	50 (25.0)
• GP***	50 (25.0)	50 (25.0)
• Nursing home****	50 (25.0)	50 (25.0)
Department (n,%)		
• Geriatrics	57 (28.5)	50 (25.0)
• Internal medicine	49 (24.5)	47 (23.5)
• Surgery	30 (15.0)	17 (8.5)
• Neurology	21 (10.5)	19 (9.5)
• Gastroenterology	17 (8.5)	22 (11.0)
• Lung medicine	17 (8.5)	21 (10.5)
• Cardiology	9 (4.5)	24 (12.0)
Clinical Frailty score (mean, SD)	N/A	5.1 (1.7)
Number of positive VMS items (mean, SD)	2.2 (1.0)	2.0 (1.0)
• Risk of delirium (n,%)	172 (86.0)	171 (85.5)
• Functional impairment (n,%)	92 (46.0)	73 (36.5)
• Risk of falling (n,%)	107 (53.5)	100 (50.0)
• Malnutrition (n,%)	56 (28.0)	49 (24.5)

*Referral = Letter sent from the GP to the hospital

**ED = Letter from the ED upon hospitalization

***GP = Clinical discharge letter from the hospital sent to the GP

****Nursing home = Clinical discharge letter from the hospital sent to the ECP in nursing home

Abbreviations: n = number of patients, %=percentage, SD = standard deviation, N/A = not applicable (no CFS scores were available during the first measurement), VMS score = (Safety Management System to detect frailty, mandatory by the Health and Youth care inspectorate)

### Primary outcome – hospital letters

Before implementation, frailty was mentioned in 12.7% of the letters from the hospital, this increased, although not significantly, to 15.3% after implementation (table 2a, *p* = 0.09). The geriatrics department mentioned frailty in the letters most often, 59.6% before and 66.0% after (supplementary Table 1). All other departments mentioned it in less than 15% of the patients, apart from lung medicine which reached 29.4% before the implementation. In a sensitivity analysis, no relationship between level of frailty and handover of frailty was found.

**Table 2a:** Primary outcome: information transfer of frail older patients from the hospital*

	Before implementation n = 150	After implementation n = 150	*p* value
Mention of frailty in letter (n,%)			
• Complete	19 (12.7)	23 (15.3)	0.09
• Incomplete	27 (18.0)	14 (9.3)	
Medication list in letter (n,%)			
• Complete list	134 (89.3)	141 (94.0)	0.20
• Incomplete list	9 (6.0)	3 (2.0)	
Resuscitation order mentioned in letter (n,%)			
• Complete	89 (59.3)	86 (57.3)	0.77
• Incomplete	2 (1.3)	1 (0.7)	
Missing letters from the patient files (n,%)			
• Missing	3 (2.0)	2 (1.3)	

*Discharge letters from the ED to the GP, from the hospital ward to the GP, from the hospital ward to the ECP

The medication list was complete in 89.3% before and in 94.0% after of the hospital letters but did not show a significant improvement (*p* = 0.20). The automated medication list was mentioned in the letters over 70% in all the departments (supplementary Table 2).

The mention of a complete resuscitation order in the discharge letter from the hospital did not change over time, 59.3–57.3% (*p* = 0.77). The mention of resuscitation order had a wide range of percentages between the departments (supplementary Table 3).

### Primary outcome – GP referral letters

The number of present referral letters from the GP did improve significantly from 32.0 to 54.0% (*p* = 0.03, table 2b). In the referral letters of these frail older patients, frailty was only mentioned in 12.5% before implementation and 7.4% after (*p* = 0.58). The handover of the medication list was 93.8% before and 100% after (*p* = 0.19) and complete resuscitation orders were present in 18.8% before and 22.2% after (*p* = 0.91).

**Table 2b:** Primary outcome: information transfer of frail older patients by referral letter from the GP

	Before implementation n = 50	After implementation n = 50	*p* value
Referral letters present the patient files (n,%)			
• Present	16 (32.0)	27 (54.0)	0.03
Mention of frailty in letter when letter was present* (n,%)			
• Complete	2 (12.5)	2 (7.4)	0.58
• Incomplete	-	-	-
Medication list in letter *(n,%)			
• Complete list	15 (93.8)	27 (100)	0.19
• Incomplete list	-	-	
Resuscitation order mentioned in letter* (n,%)			
• Complete	3 (18.8)	6 (22.2)	0.91
• Incomplete	1 (6.3)	1 (3.7)	

*Percentages are shown as total of present letters, not investigated patients

### Secondary outcomes

The discharge letter was sent within 24 h in 56.5% before and 61% after the implementation (*p* = 0.36), but large differences were seen between departments (Table 3, supplementary Table 4). The automated medication list was sent in 54.5% before and 60% after implementation (*p* = 0.27).

**Table 3:** Secondary outcome: information transfer of frail older patients

	Before implementation n = 200	After implementation n = 200	*p* value
Discharge letters sent within 24 h (n,%)	113 (56.5)	122 (61.0)	0.36
Automated medication list present* (n,%)	109 (54.5)	120 (60.0)	0.27

*Medication list is defined as an overview of current medication, which is automatically generated from the hospital file

## Discussion

The implementation of an RTA regarding the information transfer of frail older patients improved the number of GP referral letters present in the hospital electronic patient files. However, it did not lead to other significant improvements in communication between the hospital and the GP’s. The department of geriatrics generally had the highest adherence to the RTA within the hospital.

This study shows that the communication between physicians is often lacking important information. Frailty was only mentioned in the hospital letters in 15% of frail older patients. The knowledge that the patient is frail was available in the hospital, yet it was not handed over to other physicians taking care of this patient in primary care. It is known that the communication between physicians is not always optimal [22], causing information to be lost, but it is disappointing that even after an implementation program this could not be improved. The resuscitation orders were handed over better, with 57.3% present in the letter after implementation and the medication list was complete in most patients. However, though significantly improved after implementation, in only 54% of hospitalized patients we could find a referral letter by a GP in their file. Lack of information transfer could lead to worse health outcomes in patients, therefore it is important to share our experiences after out attempt to improve communication.

It is well known that a new protocol can be hard to implement and is therefore not always successful [23]. In a recent article by Auener et al [13] the possible barriers in implementing an RTA in the field of chronic heart failure was investigated. Through interviews it became apparent that the development of an RTA was relatively easy, which is an experience we share. However, several barriers were identified, such as education, referral process, relationships between healthcare providers and electronic health record systems. These are similar to the barriers we hypothesize have played a factor in the implementation problems of our RTA. Furthermore, one or more of the following factors could have influenced the implementation methods: perceived benefit of the implementation, self-efficacy, adaptability, organizational norms regarding change, training and technical assistance [16, 24–26].

First, physicians need to understand why the protocol is important for the care of frail older patients. To implement the RTA physicians were informed by an instruction handbook, a video, posters, newsletters and a presentation at every department, still the implementation was not a complete success. The presentation could perhaps be improved to be more effective. Our implementation was hampered by the work load due to the covid-19 pandemic, rapid rotations of the doctors caring for these patients (making repetition of the presentations necessary) and a high percentage of doctors being unable to work due to illness, causing knowledge about the RTA to be lost. Secondly, the willingness of the physicians is also extremely important for a successful result. Feelings regarding collaboration between medical specialist and GP’s have shown to hamper collaboration before [14]. Thirdly, the system should efficiently and sufficiently support the protocol, for example by automated information transferred into discharge letters. Finally, in the out of hours practice of the GP it is not always possible to send electronic referrals. This leads to paper referral letters brought with the patient to the ED and sometimes getting lost.

Communication about medications across transitions of care has also been known to cause difficulties previously [27]. A systematic review shows that the communication was often found to be ineffective. In our study the GP referral letters showed excellent handover, while the automated handover from the hospital to the pharmacy happened only in 60% of patients.

A future opportunity is that in our region we have chosen to also implement an RTA on the information transfer by nurses, this could help to make sure less information is lost when it is handed over from several sources. In a study performed by Olsen et al [28] in Norway it was shown that only 1% of older patients from nursing home have a nurse handover when admitted to the hospital, and 69% when discharged back. This calls for active guidelines to ensure the exchange of written information, not only from physicians, but also from nurses.

We will continue to re-implement, evaluate and strive to keep improving our results. With repetition of the education of doctors, multidisciplinary training, the possibility to report transmural incidents and trying to improve the automated generation of letters we hope to keep getting better. The lessons learned during this study could also apply to other hospitals trying to implement RTA’s.

This study did have limitations. It only used the data of the letters of one hospital and the GP’s, but did not look at how the other care facilities succeeded at the implementation of the RTA such as the nursing homes. Information might have been missed due to the retrospective design, such as the possibility to find the ‘missing’ GP letters. It is not known whether some of these patient arrived with a paper referral letter to the ED and was not scanned into the patient records, or whether these letters were not present at all.

A strength is that the inclusion was made by randomly taking patients from the pool of possible patients to eliminate the chance of selection bias. The study did look at multiple departments within the hospital to collect the data which makes it more generalizable. Furthermore, a large sample of all correspondence (14%) was assessed, which is a substantial representation of letters within our hospital.

## Conclusion

The RTA improved the presence of referral letters by GP’s to the hospital, however the primary and secondary outcomes did not improve significantly enough to evaluate the impact of the protocol on health outcomes. The implementation of the RTA needs to be continuously evaluated and improved using a plan-do-check-act cycle. After the adjustments, the protocol needs to be re-implemented and if it is more successful, the health outcomes can be evaluated.

### Electronic supplementary material

Below is the link to the electronic supplementary material.

Supplementary Material 1

Supplementary Material 2

## Data Availability

The datasets used during the current study are available from the corresponding author on reasonable request.

## List of Abbreviations

CFS: Clinical Frailty Scale
ECP: Elderly Care Physician
ED: Emergency Department
GP: General Practitioner
RTA: Regional transmural agreements
VMS: ‘Veiligheismanagementsysteem’ (Safety Management System)
