# Supplementary material for: The effect of regional transmural agreements on the information transfer of frail older patients
Source: BMC Geriatr. 2023 Nov 29;23:787. doi: 10.1186/s12877-023-04519-4 (PMC10688100; doi:10.1186/s12877-023-04519-4)
Supplement: Supplementary file 1 — Supplementary Material 1 [file 12877_2023_4519_MOESM1_ESM.docx]

**Supplemental tables**

| Table S1. Mention of frailty* | | | |  | |  | |
| --- | --- | --- | --- | --- | --- | --- | --- |
|  | Before implementation  N = 200 (%) | After implementation  N = 200 (%) |  |  |  | |  |
| Total |  |  |  |  |  | |  |
| Department |  |  |  |  |  | |  |
| - Geriatrics | 34 (59.6) | 33 (66.0) |  |  |  | |  |
| - Cardiology | - | 2 (8.3) |  |  |  | |  |
| - Surgery | 3 (10.0) | - |  |  |  | |  |
| - Gastroenterology | 1 (5.9) | - |  |  |  | |  |
| - Internal medicine | 2 (4.1) | 2 (4.3) |  |  |  | |  |
| - Lung medicine | 5 (29.4) | 2 (9.5) |  |  |  | |  |
| - Neurology | 3 (14.3) | - |  |  |  | |  |
| *Complete and incomplete included  ^a^% is based on the total amount of participants in that department group or type of letter group before or after the implementation (table 1)  .  . | | | | | | |  |

| Table S2. Mention of medication list* | | |  | | | |  |
| --- | --- | --- | --- | --- | --- | --- | --- |
|  | Before implementation  N = 200 (%) | After implementation  N = 200 (%) | |  |  |  | |
| Total |  |  | |  |  | |  |
| Department |  |  | |  |  | |  |
| - Geriatrics | 41 (71.9) | 43 (86.0) | |  |  | |  |
| - Cardiology | 9 (100.0) | 21 (87.5) | |  |  | |  |
| - Surgery | 23 (76.7) | 14 (82.4) | |  |  | |  |
| - Gastroenterology | 15 (88.2) | 17 (77.3) | |  |  | |  |
| - Internal medicine | 37 (75.5) | 38 (80.9) | |  |  | |  |
| - Lung medicine | 16 (94.1) | 21 (100.0) | |  |  | |  |
| - Neurology | 18 (85.7) | 17 (89.5) | |  |  | |  |
| *Complete and incomplete included | | | | | | | |

| Table S3. Discharge letters sent within 24 hours | | | |  |  |
| --- | --- | --- | --- | --- | --- |
|  | Before implementation  N = 200 (%) | After implementation  N = 200 (%) |  |  |  |
| Total |  |  |  |  |  |
| Department |  |  |  |  |  |
| - Geriatrics | 32 (56.1) | 28 (56.0) |  |  |  |
| - Cardiology | 9 (100.0) | 17 (70.8) |  |  |  |
| - Surgery | 12 (40.0) | 7 (41.2) |  |  |  |
| - Gastroenterology | 12 (70.6) | 14 (63.6) |  |  |  |
| - Internal medicine | 22 (44.9) | 28 (59.6) |  |  |  |
| - Lung medicine | 13 (76.5) | 12 (57.1) |  |  |  |
| - Neurology | 13 (61.9) | 16 (32.0) |  |  |  |
| *Complete and incomplete included | | | | | |

| Table S4. Mention of resuscitation order* | | | |
| --- | --- | --- | --- |
|  | Before implementation  N = 200 (%) | After implementation  N = 200 (%) |  |
| Total |  |  |  |
| Department |  |  |  |
| - Geriatrics | 38 (66.7) | 35 (70.0) |  |
| - Cardiology | 7 (77.8) | 7 (29.2) |  |
| - Surgery | 5 (16.7) | 4 (23.5) |  |
| - Gastroenterology | 9 (52.9) | 6 (27.3) |  |
| - Internal medicine | 9 (18.4) | 13 (27.7) |  |
| - Lung medicine | 12 (70.6) | 17 (81.0) |  |
| - Neurology | 15 (71.4) | 12 (63.2) |  |
|  | | | |
